# Application of Hierarchical/Multilevel Models and Quality of Reporting (2010–2020): A Systematic Review

**DOI:** 10.1155/2024/4658333

**Published:** 2024-03-08

**Authors:** Killian Asampana Asosega, Atinuke Olusola Adebanji, Eric Nimako Aidoo, Ellis Owusu-Dabo

**Affiliations:** ^1^Department of Statistics and Actuarial Science, Kwame Nkrumah University of Science and Technology, Kumasi, Ghana; ^2^Department of Mathematics and Statistics, University of Energy and Natural Resources, Sunyani, Ghana; ^3^Department of Global and International Health, Kwame Nkrumah University of Science and Technology, Kumasi, Ghana

## Abstract

**Introduction:**

Multilevel models have gained immense popularity across almost every discipline due to the presence of hierarchy in most data and phenomena. In this paper, we present a systematic review on the adoption and application of multilevel models and the important information reported on the results generated from the use of these models.

**Methods:**

The review was performed by searching Google Scholar for original research articles on the application of multilevel models published between 2010 and 2020. The search strategy involved topics such as “multilevel models,” “hierarchical linear models,” and “mixed models with hierarchy.” The search placed more emphasis on the application of hierarchical models in any discipline but excluded software methodological development and related articles.

**Results:**

A total of 121 articles were initially obtained from the search results. However, 65 articles met the inclusion criteria for the review. Out of the 65 articles reviewed, 46.2% were related to health/epidemiology, 15.4% to education and psychology, and 16.9% to social life. The majority of the articles (78.5%) were two-level models, and most of these studies modelled univariate responses. However, the few that modelled more than one response modelled them separately. Moreover, 83.1% were cross-sectional design, and 9.2% and 6.2% were longitudinal and repeated measures, respectively. Moreover, a little over half (55.4%) of articles reported on the intraclass correlation measure, and all articles indicated the response variable distribution where most (47.7%) were normally distributed. Only 58.5% of articles reported on the estimation methods used as Bayesian (20%) and MLE (18.5%). Again, model validation measures and statistical software were reported in 70.8% and 90.8% articles, respectively.

**Conclusion:**

There is an increase in the utilization of multilevel modelling in the last decade, which could be attributed to the presence of clustered and hierarchically correlated data structures. There is a need for improvement in the area of measurement and reporting on the intraclass correlation, parameter estimation, and variable selection measures to further improve the quality of the application of multilevel models. The integration of spatial effects into multilevel models is very limited and needs to be explored in the future.

## 1. Introduction

Multilevel analysis is a collection of statistical techniques used to examine the relationship between variables characterizing individuals and those characterizing groups [1]. Multilevel modelling usually has to do with data that are hierarchically structured in nature. In multilevel modelling, related research variables can be defined and declared at any level of the hierarchy based on the focus and data structure. While some variables can be measured at their own natural level, others have to be moved from one level to another by either aggregation or disaggregation [1]. In multilevel modelling, the finer scale at which the response or dependent variable is measured is called the lower level, whereas the aggregate scale is referred to as the higher level. Multilevel models usually anticipate both differences between the higher-level units and dependence within those units. Within observations, dependences are mostly anticipated because their members are assumed to be influenced by the same aggregate effects [2]. Moreover, the existence of group dependence among lower-level units violates the fundamental assumption of independence in a standard regression analysis, resulting in an increase in the risk of inefficient model estimation and inappropriate inferences with respect to parameter estimates [2–4].

Multilevel models, also known as hierarchical linear models (HLM), have in recent times received attention and utilization from several disciplines, especially in the social, educational, biological, and medical fields where datasets are usually nested [5]. Hierarchical linear models are also very useful in longitudinal data structures, where measurements measured at different points in time are nested within the observations or units on which those measurements were made. Modelling of the outcome variable in these situations presents a flexible way to appropriately capture and account for the nested data structure to ensure that standard errors and model parameters are accurately estimated [4, 6].

A study by El-Horbaty and Hanafy [7] argued that most problems in social sciences often involve investigations of the relationship between individual and society-level characteristics. The general notion is that individuals and their social groups are conceptualized as a hierarchical system, where the individuals and groups are defined at separate levels of this hierarchical system. There exist several forms of hierarchical models, which vary in terms of the type of design (random intercept, random slopes, and random coefficients regression model), number of levels, measurement scale of the dependent variable (continuous and categorical), and number of measured responses (univariate and multivariate).

Hierarchical analyses are performed on data that have some form of nested structure. Data with nested structures are often associated with some form of dependency. For instance, in a corporate working environment, individuals in the same department or unit often showcase similar performance and provide similar responses to questions about aspects of the work environment. According to [8], the presence of nonindependence in any given dataset may be considered either a nuisance variable or something to be fundamentally appreciated, but the prevalence and growing presence of nested data require a variety of statistical tools to easily handle nested data.

The term “multilevel analysis” has been used most often to describe the set of statistical analyses, also referred to as random coefficient models, random-effects, and mixed-effects models [9–11]. Hierarchical models are generally suitable for dealing with nonindependence.

Several studies [12–14] have shown that most geographical processes or events often display strong differences between locations or regions. Empirical results from physical geography have established that this form of *spatial heterogeneity* has long been known as very vital to the accuracy of models [15]. According to Browne and Goldstein [12], the most effective way to address spatial heterogeneities that have been observed extensively in geography as well as other areas where clustered or hierarchical structured data are observed is multilevel modelling. In a related study, Gelman [16] also observed that random-effects models are very desirable across several disciplines of science precisely due to their ability to explain between and within-group heterogeneity.

Again, Wolf et al. [17] emphasized that multilevel models have been used for quite a long time across a variety of geographical problems. For instance, related studies in epidemiology employ multilevel models to estimate the effect between conditions such as smoking, asthma, mental health, and cancer [18–21]. Moreover, multilevel models are used in areas such as economic and urban structure analysis [22–24]. In economics, multilevel models have been used to highlight the impact of economic and individual indicators on the well-being of citizens [25], country and industry factors on job demand [26], and on privatization of state enterprises [27]. Multilevel models have also been utilized to examine country- and individual-level influences on sanitation and environmental-related issues [28, 29].

In real life problems, the usual assumption of statistical independence of observations does not hold for nested structured data or data with an underlying hierarchy. More often than not, however, the populations from which these data are collected or generated usually have multifaceted structures where measurements on data units are not mutually independent but depend on each other through some form of underlying complex structural relationships. These multifaceted structured relationships cannot be effectively captured and accounted for by models that assume independence of sampled observations from a given population [1, 30, 31].

Methodological details in scientific research are very important as they have a significant impact on the validity of statistical results and inferences arising from the generated results. Hence, insufficient methodological details in scientific articles have significant setbacks in terms of study replication, inaccurate parameter estimates, and subsequent invalid inferences. For instance, according to Hox et al. [32], analysis of variables at the wrong level leads to statistical and conceptual problems, which affect the statistical power of the analysis performed. In addition, insufficient methodological details particularly in multilevel modelling could result in ignoring the nested structure of datasets and could yield inaccurate and imprecise parameter estimates and their corresponding standard errors [33, 34]. Lack of sufficient details of the methodology used could affect the trustworthiness of study findings [4].

Despite the continuous increase in utilization of multilevel models across diverse fields of endeavour, there are no clear standardized directions and guidelines on the usage and reporting of the generated results. Moreover, there is limited review of literature on the development and applications of multilevel models in the last decade, a gap this study seeks to bridge.

This study seeks to provide the current update by means of a systematic review of some relevant information on the use of multilevel models in the existing literature in the last decade (2010–2020) reported across several areas of application. The time scale was purposely chosen to understand how reporting standards in the utilization of multilevel models have been and evolved over the period which will inform and direct further review in subsequent decades. Moreover, the review will help identify the limitations and strengths of these models in the literature which could be leveraged upon to improve the quality of statistical information reported in the use of multilevel models.

Multilevel models are accredited and desirable across a wide range of disciplines due to their ability to account for group heterogeneity [16]. Multilevel models further acknowledge correlations in observations within the same group which is popularly known in geography as platial or horizontal dependence [35]. However, platial dependence for correlation among observations from the same place is different from the Anselin [36] spatial dependence concept which accounts for the fact that nearby observations are more related irrespective of regional boundaries [37]. Accounting for both spatial and platial dependence in one model results in improvements in the precision and accuracy of parameter estimates and enhances the validity and reliability of inferences [35, 38]. Also, Wolf et al. [17] highlight the powerfulness of multilevel models to account for spatial dependence. They further recommend the blend of spatial and platial effects to maximize the advantages presented in both. Generally, MLMs are utilized to yield narrow interval estimates with some biasedness, whereas spatial models produced unbiased estimates but wide intervals, and hence, the integration of both multilevel and spatial effects provides improvements in results generated. Moreover, ignoring spatial effects in multilevel models could result in more extreme estimates but narrower confidence intervals. This stems from the underestimation of standard errors which could lead to serious consequences as nearby spillover effects could remain unaccounted for in the model [32, 39].

## 2. Methods

This systematic review was carried out in accordance with the Preferred Reporting Items for Systematic Reviews and Meta-analysis (PRISMA) statements [40, 41]. This study further reported the review in line with the PRISMA guidelines.

This review considered original articles published between 2010 and 2020 inclusive, and the search was performed using Google Scholar. The search strategy adopted the following topics: “hierarchical linear model,” “multilevel model,” and “mixed model.” However, the area of application of these models was not restricted.

### 2.1. Selection of Studies Included in the Review

Articles obtained from the search were eligible for inclusion if they were original research articles and must have been written in English peer-reviewed journals reporting on the application of multilevel models. Articles on statistical development of the methodology were also excluded from the review.

### 2.2. Identification of Studies

**Figure 1:**
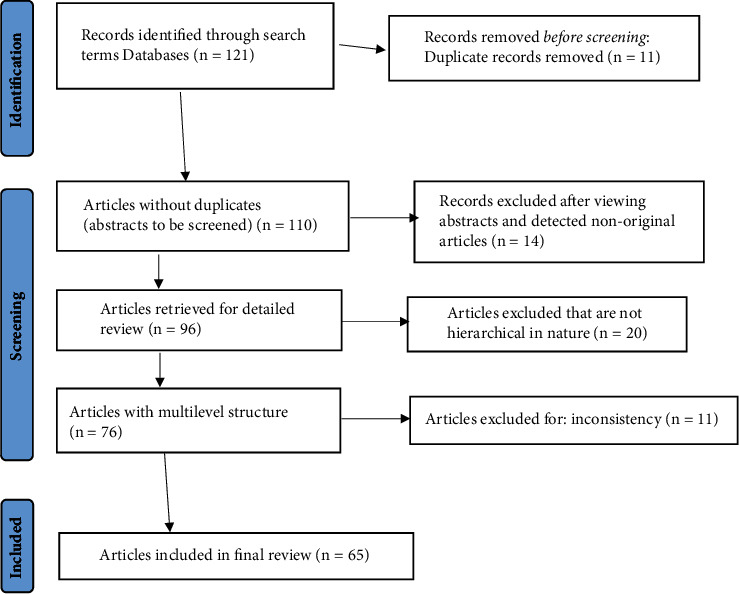
Flowchart for the selection of articles. Source: BMJ 2021; 372: n71. doi: 10.1136/bmj.n71. https://www.prisma-statement.org/.

The selected articles for the review were used to extract some information presented in Tables 1 and 2. The PRISMA flowchart (Figure 1) was used to summarize all article selection process [40, 41]. During the first review phase, a total of 121 articles were identified, out of which 11 were duplicates. Moreover, after a careful examination of the abstracts of the selected studies, some were excluded because they were nonoriginal articles (conference proceedings) and those that did not have either multilevel or hierarchical modelling as a keyword or abstract or in the title of the article.

In the second phase of the review, out of the 96 articles considered, only 76 pertained to multilevel/hierarchical modelling applications to hierarchically structured data. Hence, 11 articles were further excluded due to nonexistence and inconsistency.

The third aspect of the review looked at the full-text versions of the eligible articles included in this review. A comprehensive review of the 65 articles was conducted with emphasis on the number of levels, number of observations at the lowest and highest levels, method of estimation, method of model validation, presence of outliers in datasets, measurement of intraclass correlation (ICC), number of response variables, presence of spatial effects, and software (packages/macros) used, and others are discussed in the results section of this review.

### 2.3. Study Characteristics

For the study design, the review considered several aspects of each article. Some of the key aspects include the number of levels in the hierarchy, estimation method, sample sizes at both the lowest and highest levels of the hierarchy, type of response variable number or response variables, type of study, data source and sampling techniques used, model validation measures, presence of outliers and how they are handled, and measurement of ICC which is very fundamental in hierarchical modelling framework.

In addition, the review also looked at whether or not the distribution of the response variable was indicated as well as assumptions concerning the MLM and whether those assumptions were met or not based on empirical statistics presented in the articles selected for this review. The study also investigated whether spatial components were included in the multilevel and how they were captured in the modelling framework to help direct future focus on the topic.

### 2.4. Inferential Information on Studies

This section presents some important inferential indicators concerning multilevel models in general. The hierarchical model is characterized by random and fixed effects or components, where the random components are usually associated with grouping (higher)-level variables within the hierarchy which account for the grouping-level variables in the model.

Moreover, the review reported on the estimation techniques as well as the software employed in each selected study since these usually have effects on the validity of parameter estimates and inferences [42, 43]. Besides, the computational times, flexibility, and user-friendliness of these software programs vary considerably. Several software programs are available for implementing hierarchical modelling. These programs include SPSS (version 19 and above) which includes a GLMM framework in the GENLINMIXED procedure [44], SAS macro GLIMMIX which was the very first software introduced for HLM based on the penalized quasi-likelihood (PQL) estimation procedure, lme4 [45], glmml, MASS(glmm PQL functionality), most recent sjstats [46], R2jags in *R*, and xtmixed and gllamm functions in STATA [47, 48].

The significance or hypothesis testing regarding the fixed and random effects in multilevel models is usually tested and assessed separately. The fixed-effects components of MLM are usually assessed by the Wald test, while those of the random-effects variances are tested through LRT or by comparing the goodness-of-fit measures of the models using the Bayesian and Akaike information criteria [49].

The variable selection strategy used in the respective studies selected under review is also essential and worth noting and considering. The most commonly used variable selection is the stepwise (forward or backward) selection strategy. This is usually based on the model fit indices such as AIC, BIC, or DIC and the Wald test on model parameters to examine their respective contributions in the fitted models. Notwithstanding, some models do not employ any variable selection procedure as in the case of confirmatory analysis where a specific hypothesized model of interest is fit, usually based on previous research or recommendations from theory and experience [33].

## 3. Results

The development and application of multilevel models across several disciplines have received and continuous growing interest in recent times in the scientific literature. From the initial 121 articles obtained from the search, the detailed results presented are regarding the 65 articles included in the final detailed review.

**Figure 2:**
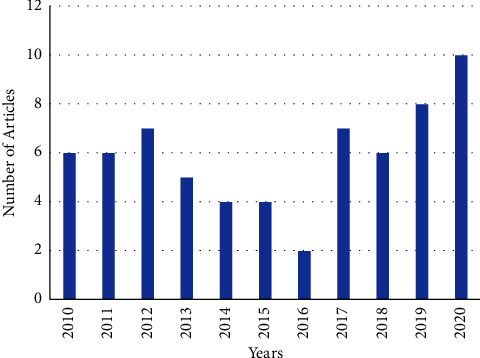
Number of reviewed articles by years.

Out of the final sixty-five (65) articles, the highest and second highest of 10 (15.4%) and 8 (12.3%) of the articles were published or recorded in 2020 and 2019, respectively (Figure 2). Thirty articles (46.2%) were applied in health/epidemiology, 11 (16.9%) in social life, 10 (15.4%) in education/psychology, and the least of 1 article in the area of climate science. On the number of levels in the multilevel structure of the models used, 51 articles (78.5%) were at 2-level models and 13 (20.0%) were 3-level hierarchical models. Moreover, over eighty percent were cross-sectional studies, whereas 15.4% (10 articles) were longitudinal/repeated measure studies, and the only article was an experimental study.

A total of 55 articles (84.6%) reported on the sample sizes at the lowest level (level-1) of the multilevel structure, which ranged from 6 to 5,700,000 observations with a corresponding median of 7,079 (*Q*1 = 686; *Q*3 = 44,906). With respect to 2-level multilevel models, 46 (90.2%) articles reported the sample size at the lowest level, which ranged from 244 to 5,700,000 (*Q*1 = 1077; *Q*3 = 49,062), while the 3-level hierarchical model recorded sample sizes ranging from 6 to 231, 326 with a median of 2,498 (*Q*1 = 59; *Q*3 = 17,296) observations at the lower level.

The lower-level units in the reviewed articles selected mostly (69.2%) referred to individuals (such as patients, customers, students, nurses, employees, and women) irrespective of the number of levels in HLM. The number of response variables in the selected studies varied from one (1) response (*n* = 58; 89.2%) to 4 (3.1%) response variables. However, all two or more response variables in the articles were modelled separately as univariate variables one after the other rather than as a multivariate variable. The distribution of the response variables was reported in all articles as normal (*n* = 31; 47.7%), binomial/binary (*n* = 24; 36.9%), ordinal (*n* = 5; 7.7%), normal and binary (*n* = 2; 3.1%), and one (1) each for ordinal and binomial, Poisson, and multinomial distributions as available software can fit or model a variety of distributions as response variables.

For the estimation method used in each of the reviewed articles, the maximum likelihood (MLE) was implemented in 12 articles: Bayesian through MCMC (*n* = 13), restricted maximum likelihood (ReML; *n* = 3), generalized least squares (GLS, *n* = 2), weighted least squares (WLS, *n* = 2), ReML and MLE (*n* = 3), ReML and Bayesian (*n* = 1), and generalized estimation equations (GEE; *n* = 1). These estimation techniques were mostly implemented in *R* (*n* = 13), SAS (*n* = 9), Stata (*n* = 9), Mlwin (*n* = 2), HLM (*n* = 6), SPSS (*n* = 5), MPlus (*n* = 5), and Stata and Mlwin (*n* = 2). Articles in which *R-software* was used employed packages such as lme4, Sjstats, runjags, lmer, spdep, Jags, and HSAR. For SAS, the packages used included Glimmix and PRO MIXED, and in Stata, glamm, xtlogit, and xtmixed were used.

Moreover, 55.4% of the articles selected presented intraclass correlation (ICC) measures, which quantify the group (cluster)-level effects on the response measured at the lowest level. Model validation (goodness of fit) measures were indicated in 45 (73.8%) articles using indices such as Akaike information, Bayesian information, deviance information, quasi-likelihood information criteria, and likelihood ratio test (LRT) with some reporting at least one of these fit indices. Spatial effects were considered in only 4 (6.6%) out of the 61 articles reviewed. Moreover, the presence of outliers was reported in 3 articles whereby one of such removed the outliers before model fitting.

## 4. Discussion

Multilevel models have in recent times gained popularity across several disciplines such as geography [12, 13], education and psychology [50–53], sports [54, 55], public health and epidemiology [56–60], and economics [26, 38, 61]. However, the application of these models is somehow scarce or nonexistent in engineering and climate science as observed in this review. According to Casals et al. [33], the validity of conclusions and or inferences in any scientific paper is associated with the appropriateness of the statistical methods employed to obtain the results. Therefore, the relevance to adequately describe the statistical techniques used in the analysis which accords the reader the opportunity to determine whether the conclusions and inferences made are right or otherwise. As evident in the results, most of the important information on HLMs as recommended in [49, 62] was not reported or presented which consequently undermines the reliability of the generated results as well as the validity of conclusions in such studies. For instance, nearly half (42.6%) of the articles selected failed to report on the estimation procedure used in generating the results. Some also did not report on the software used for the analysis of data.

There are divergent views on the issue of sample size per cluster and their impact on multilevel models. For example, Clarke [63] opined that cluster sizes below five per cluster result in overestimation of cluster-level variance in 2-level MLMs but recommend a mean of five observations per cluster to yield more valid and reliable parameter estimates. However, Hox and McNeish [39] argue that the smaller cluster sizes are problematic but often dissipate with a large number of clusters. Regarding parameter estimation, the maximum likelihood (ML) estimation and weighted least squares (WLS) are efficient in large sample cluster sizes, whereas the Bayesian method is efficient and recommended for small sample sizes to ensure precision in estimates. Despite the nonexistent clear rule on the minimum sample size requirement, recommendations vary based on the complexity of the model and intra-class correlation. Hox and McNeish [39] further opined that accurate parameter estimates and standard errors linked with few clusters could be obtained by using restricted maximum likelihood estimation instead of full MLE.

Besides the impact of small sample sizes on parameter estimates and standard errors, small samples also have significant effects on the statistical power of tests as the result in low power to reject an incorrect null hypothesis [64]. The different recommendations on cluster sizes include Kreft's [65] minimum of 30 observations and 30 groups, Maas and Hox [66] more than 30 groups and less than 30 observations, Hox's [1] at least 20 observations for 50 groups, Clarke and Wheaton [67] minimum of at least 10 observations per cluster and at least 200 clusters to generate unbiased and efficient estimates, and Snijders and Bosker [68] suggestion of at least one observation per group with some groups with larger sizes. Vallejo et al. [69] highlight the importance of a larger number of groups than a larger number of observations per group and recommend at least 50 groups with at least 20 observations per group to produce robust and efficient estimates. These different suggestions on the number of groups and observations per group underscore the need for standardization of these areas to enhance the validity and reliability of generated results in the utilization of MLMs.

Moreover, the inferential information such as confidence interval estimation, hypothesis testing, and model validation measures are usually related to the estimation technique used. Therefore, the absence of information regarding the estimation method used often results in the difficulty for readers to evaluate the methods used for the inferences and conclusions. Again, some estimation methods have limitations depending on the situation as in the case of MLE which produces biased and inconsistent parameter estimates when small sizes are small and for the PQL, which also generates biased parameter estimates when the sample standard deviations of random components are large in binary data [49].

In addition, most of the articles, besides failing to report on the estimation procedure, did not measure the intraclass correlation (ICC), which is very fundamental and important in the multilevel modelling framework. In [70–72], the value of the ICC indicates the existence of a multilevel structure and therefore recommends multilevel modelling to adequately account for clustering effects which could result in underestimation of standard errors and subsequent loss of statistical power when ignored. The absence of the ICC in a study to quantify the level of clustering effects could undermine results and findings since the ICC is very fundamental in informing the use of multilevel models. Moreover, failure to report the ICC value could affect the trustworthiness of findings. According to Merlo et al. [73], without the ICC value, the degree of clustering effect may not be ascertained. In Hayes [74], quantifying the group-level effect in terms of ICC is a basic requirement and recommends the implementation of MLMS with at least an ICC of 0.05. Based on Hayes' suggestion, ICC values in studies with clustering effects should be very fundamental to ensuring transparency and reproducibility.

Model validation and selection indices were not reported in some articles and therefore can affect the trustworthiness of findings in such articles. Casals et al. [33] further confirmed that fulfilment of model assumptions results in valid statistical inferences of generated results and conclusions. It is therefore imperative for authors to report on these model assumptions and validation measures as well as how they work and are assessed for replication and reproducibility of these studies.

## 5. Conclusion

The application of hierarchical models over the years has increased significantly across a wide spectrum of areas, most especially in public health [56–58, 60, 75], education and psychology [50–53], and geography, economics, and development [26, 38, 43, 61]. This is a result of the underlying hierarchical structure observed in most available data in these areas [1, 5, 35].

This review shows an increase in the adoption of multilevel models across a wide range of disciplines, which could be attributed to the presence of clustered surveyed data [1, 5, 35]. However, the review observed a low reportage of the clustering/grouping effect in terms of the ICC or variance partition coefficient which validates the application of multilevel models. Again, the method of parameter estimation and model adequacy/assessment are not clearly indicated to ensure that the underlying assumptions are met or otherwise for reproducibility. We recommend the standardization of reporting guidelines aimed at improving the quality of reporting and validity of findings among researchers and editors [76].

## 6. Limitation

The review conducted encountered a few limitations which include (1) the possibility of potential bias as a result of search terms used in searching for articles might have been very sensitive and therefore could have ignored some quality-related articles and (2) the relatively small number of articles selected during this review in the 11-year period.

## Figures and Tables

**Table 1:** Characteristics of reviewed articles.

Area of application	*n* = 65
Climate science	1 (1.5%)
Economics	6 (9.2%)
Education/psychology	10 (15.4%)
Health/epidemiology	30 (46.2%)
Human development	2 (3.1%)
Planning	2 (3.1%)
Social life	11 (16.9%)
Sports	3 (4.6%)
Number of levels	
Two	51 (78.5%)
Three	13 (20.0%)
Four	1 (1.5%)
Number of response variables	
One	58 (89.2%)
Two	4 (6.2%)
Three	1 (1.5%)
Four	2 (3.1%)
Lower-level units	
Individuals/human	45 (69.2%)
Location	5 (7.7%)
Time period	3 (4.6%)
Firms	2 (3.1%)
Publications/manuscripts	3 (4.6%)
Households	3 (4.6%)
Properties	2 (3.1%)
Measurements	1 (1.5%)
Angles	1 (1.5%)
Study design	
Cross-sectional	54 (83.1%)
Experimental	1 (1.5%)
Longitudinal	6 (9.2%)
Repeated measures	4 (6.2%)
Measurement of ICC	
Yes	36 (55.4%)
No	29 (44.6%)

**Table 2:** Characteristics of statistical inference and estimation methods.

Method of estimation	
Bayesian	13 (20.0%)
GEE	1 (1.5%)
GLS	2 (3.1%)
MLE	12 (18.5%)
PQL	1 (1.5%)
ReML	3 (4.6%)
ReML and Bayesian	1 (1.5%)
ReML and MLE	3 (4.6%)
Weighted least squares	2 (3.1%)
NI	27 (41.5%)
Distribution of response	
Binary	24 (36.9%)
Multinomial	1 (1.5%)
Normal	31 (47.7%)
Normal and binary	2 (3.1%)
Ordinal and binary	1 (1.5%)
Ordinal	5 (7.7%)
Poisson	1 (1.5%)
Model validation measures	
AIC	3 (4.6%)
BIC	2 (3.1%)
AIC and BIC	7 (10.8%)
AIC, BIC, and LRT	11 (16.9%)
DIC	7 (10.8%)
*R*-square	3 (4.6%)
LRT	5 (7.7%)
RMSEA/MSE	6 (9.2%)
HDI/QIC	2 (3.1%)
NI	19 (29.2%)
Statistical software	
HLM	6 (9.2%)
Mlwin	8 (12.3%)
Mplus	4 (6.2%)
*R*	13 (20.0%)
SAS	9 (13.8%)
SAS and Mlwin	1 (1.5%)
SPSS	5 (7.7%)
Stata	9 (13.8%)
Stata and Mlwin	2 (3.1%)
WinBUGS	2 (3.1%)
NI	6 (9.2%)
Statistical packages	
GLIMMIX	5 (7.7%)
GLLAMM	1 (1.5%)
HSAR	1 (1.5%)
JAGS (rjags, runjags, and coda)	2 (3.1%)
lme4/lmer	4 (6.2%)
lme4, spdep, and sjstats	2 (3.1%)
Nmle	2 (3.1%)
PROC MIXED/NLMIXED	4 (6.2%)
VCMMR estimation	1 (1.5%)
xtlogit	1 (1.5%)
xtmixed	1 (1.5%)
NI	41 (63.1%)
